# Lung cancer screening beliefs in Armenia

**DOI:** 10.3389/fonc.2023.1062690

**Published:** 2023-06-16

**Authors:** Nathan Chu, Haley Tupper, Tamara Galoyan, Armine Lulejian, James Dickhoner, Marine Hovhannisyan, Shant Shekherdimian

**Affiliations:** ^1^ Institute for Society and Genetics, University of California, Los Angeles, Los Angeles, CA, United States; ^2^ Department of General Surgery, University of California, Los Angeles, Los Angeles, CA, United States; ^3^ School of Education, Drexel University, Philadelphia, PA, United States; ^4^ Keck School of Medicine, University of Southern California, Los Angeles, CA, United States; ^5^ Innovation Studio, Children’s Hospital Los Angeles, Los Angeles, CA, United States; ^6^ Faculty of Public Health, Yerevan State Medical University, Yerevan, Armenia; ^7^ Department of Pediatric Surgery, University of California, Los Angeles, Los Angeles, CA, United States

**Keywords:** lung cancer, screening, health beliefs, Armenia, developing countries, smoking, screening barriers, health education (MeSH)

## Abstract

**Introduction:**

In Armenia, an upper-middle income country, 93% of deaths are from non-communicable diseases and over half of the male population smokes. Armenia has more than double the global lung cancer incidence. Over 80% of lung cancer is diagnosed at stages III or IV. However, there is a significant mortality benefit in detecting early-stage lung cancer via screening with low-dose computed tomography.

**Methods:**

This study employed a rigorously-translated, previously-validated survey based on the Expanded Health Belief Model to understand how Armenian male smokers’ beliefs would affect lung cancer screening participation.

**Results:**

Survey responses highlighted key health beliefs that would mediate screening participation. Most respondents felt they were at risk for lung cancer, but over 50% also believed their cancer risk was equivalent to (or less than) non-smokers’ risk. Respondents also overwhelmingly agreed a scan could help detect cancer earlier, but fewer agreed early detection could reduce cancer mortality. Important barriers included absence of symptoms and costs of screening and treatment.

**Discussion:**

Overall, the potential to reduce lung cancer-related deaths in Armenia is high, but there are a number of central health beliefs and barriers that would limit screening uptake and effectiveness. Improved health education, careful consideration of socioeconomic screening barriers, and appropriate screening recommendations may be useful in overcoming these beliefs.

## Introduction

Lung cancer is the second most common cancer globally (1) and almost two-thirds of lung cancer deaths occur in low and middle-income countries (LMICs) (2). Within this cohort, upper-middle income countries have the highest rates of lung cancer incidence and mortality (2). Armenia, an upper-middle income country of 3 million in the South Caucasus (3), has more than twice the global age-standardized incidence of lung cancer (4, 5) and the magnitude is only increasing (6). In 2019, Armenian men ages 60-80 had more than a 1 in 20 chance of dying from lung cancer (6). Lung cancer epidemiology already varies significantly by gender, but the contrast is even more stark in Armenia, where mortality is nearly five times higher in men (6). Disparate mortality rates are largely due to gendered tobacco use. Over half of the Armenian male population smokes, compared to 2% of women, and tobacco use is heavy (7, 8).

Primary and secondary non-communicable disease (NCD) prevention should be a priority for Armenia, where 93% of deaths are attributable to NCDs (9). Tobacco cessation is one of the most cost-effective interventions to combat NCDs, including lung cancer (10, 11). Although Armenia signed the World Health Organization (WHO)’s Framework Convention on Tobacco Control in 2004, initial movement was sluggish. More recently, the government mandated health warnings on tobacco packaging (2016), increased tobacco taxes (2019), and banned indoor smoking and public display of tobacco products (2022) (10). However, tobacco cessation services are limited, most advertising is still legal, and teenage tobacco use is increasing (10). Furthermore, Armenia does not have a national lung cancer screening program, yet detection of asymptomatic, early-stage disease requires organized screening protocols (7). Annual low-dose computed tomography (LDCT) in high-risk individuals (e.g. current and former smokers) is the only screening method proven to successfully identify lung cancer at earlier stages with a 20-26% mortality benefit (12, 13). In Armenia, in the absence of organized screening, over 80% of lung cancer cases are diagnosed at stages III or IV (7) and lung cancer is almost universally fatal (4).

**Figure 1:**
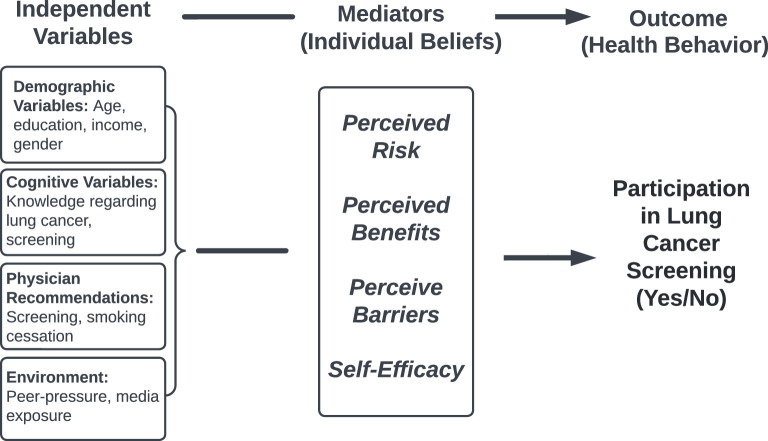
*Expanded Health Belief Model.* *Adapted figure reproduced with permission from original author Lisa Carter-Harris, PhD.

The success of any cancer screening program depends on its uptake. It has been shown in other LMICs that many cultural barriers and health beliefs exist that could potentially limit cancer screening participation, from anxiety around possible diagnosis to fear of treatment and fatalism (14, 15). Consequently, prior to piloting any potential screening programs, it is important to preliminarily understand barriers to uptake in Armenia. One of the most commonly utilized models to evaluate health-promoting behaviors, such as lung cancer screening, is the Expanded Health Belief Model (EHBM), where individual beliefs mediate participation in a health behavior. In this model, individual beliefs are deconstructed into several conceptual elements, specifically perceived risk, perceived barriers, perceived benefits, and self-efficacy (see **Figure 1**) (16). In this study, a previously-validated lung cancer screening-specific version of the EHBM was translated and employed. Given differential smoking patterns, this study focused on men. The purpose of this study was to assess the attitudes and beliefs of male smokers towards lung cancer screening in Armenia’s capital using a pre-validated instrument. Three core research questions guided the data analysis and discussion: 1) What are the perceptions of Armenian male smokers regarding lung cancer screening? 2) Is there any relationship between their perceptions of risk, benefits, barriers, and self-efficacy regarding lung cancer and screening? 3) Do their demographic characteristics impact their beliefs about lung cancer and screening?

## Methods

Institutional review board (IRB#21-000784) exemption was obtained from the Office of Human Research Protection Program at the University of California, Los Angeles. Local institutional review board approval was also obtained from the Ethics Committee at Yerevan State Medical University.

### Study population

A stratified random sampling approach was used to recruit study participants. Active or former male smokers, aged 40 to 70, were recruited proportionally from the 12 administrative districts of Yerevan, Armenia’s capital city. Armenian men were surveyed because they have significantly higher rates of smoking compared to women and are therefore at higher risk for lung cancer (6). The age criteria was determined from the United States Preventive Service Task Force’s (USPSTF) lung cancer screening age criteria of 50 to 80 years (11). The age range for recruited participants was lower than existing guidelines to account for the perspectives of those who were nearing screening age. Those with previous lung cancer diagnoses or history of LDCT were excluded from this study. To calculate our sample size target of 384 men, we used a population of 500,000 men in Yerevan with a confidence level of 95%, a margin of error of 5%, and a 50% prevalence based on the proportion of Armenian males who smoke.

### Survey design

The survey was adapted from a previously-validated lung cancer health belief scale (alpha >0.7) utilizing a four-point Likert-type scale to analyze four constructs: 1) perceived risk, 2) perceived benefits, 3) perceived barriers, and 4) self-efficacy (16). The survey was designed and administered through Qualtrics online survey software (Qualtrics, Provo, UT). The survey was translated from English to Armenian and back-translated into English to check for accuracy. Questions were added to the Armenian-language survey to account for local relevance and to gather demographic information. The survey was then administered to native Armenians to ensure face validity. Using the feedback provided, redundant questions due to translation were removed. No prior information or materials regarding lung cancer or lung cancer screening were provided to participants.

### Data collection

**Figure 2:**
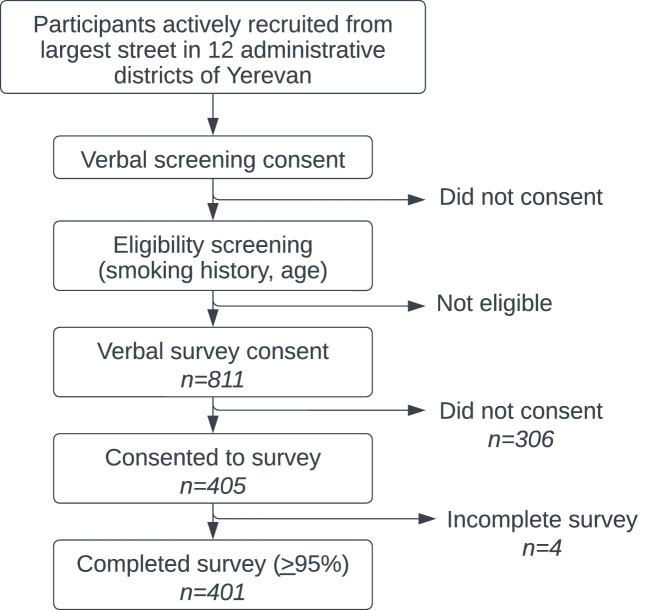
Recruitment & sampling methodology.

Data was collected proportionally based on percent of population from each of Yerevan’s administrative districts. Study participants were recruited Monday through Sunday from October 22, 2021 to January 30, 2022. Potential participants were randomly approached and recruited from the largest street in each of Yerevan’s 12 administrative districts (see **Figure 2**). Trained local surveyors first obtained consent to ask four filter questions to determine study eligibility. There was no mechanism to characterize potential participants who were initially unwilling or ineligible to participate. For eligible participants, verbal informed consent to participate in the full survey was then obtained and documented by surveyors in Qualtrics. Finally, surveyors verbally administered the survey to participants and electronically recorded the deidentified results in a mobile version of Qualtrics’ data collection software.

### Statistical analysis

The survey data was analyzed using IBM SPSS 26 Statistics software. The statistical analysis included 1) descriptive statistics reporting on frequency analysis, 2) correlational analysis reporting on Spearman’s Rho correlation coefficient, and 3) stepwise multiple linear regression analysis. Prior to the analysis, the data was screened for missing values, followed by testing assumptions, including normality, linearity, homoscedasticity, and absence of multicollinearity. Surveys that were at least 95% complete were included in the analysis. For all statistical analyses, the significance criterion was set at p <.05. Composite mean scores for each construct were calculated and used in correlational and inferential statistical analyses. Correlational analysis included exploring the relationship between different demographic variables and attitudes towards lung cancer screening, specifically the four constructs of perceived risk, perceived benefits, perceived barriers, and self-efficacy. We also examined the correlation between the responses in one construct (e.g., perceived benefits) to responses in each of the other three constructs (e.g., perceived risk, perceived barriers, self-efficacy). A forward stepwise linear regression analysis was used to examine whether participants’ demographic characteristics significantly impacted their perceived risk, perceived benefits, perceived barriers, and self-efficacy. The stepwise method in SPSS is a variation of the forward regression method except that every time a predictor is added to the equation, a removal test is made for the least useful predictor (17). Specifically, the predictor variables included self-rated health, marital status, education level, monthly income, age, and pack year (see **Table 1** for the categories listed within each predictor variable). The dependent variables included the participants’ perceived risks, perceived benefits, perceived barriers, and self-efficacy. The significance level for the variable inclusion in the regression models was set at <.05 and candidate variables were added until no further variables satisfied the entry criterion (p <0.5).

**Table 1:** Demographic characteristics of respondents.

Characteristic	n (%)
Total Respondents	n= 401
Age	
40-50 yrs	155 (38.7%)
51-60 yrs	132 (33%)
61-70 yrs	114 (28.3%)
Avg Cigarettes per Day *Median (IQR)	22 (10)
Avg Years of Smoking *Mean (SD)	25 (14)
Prior Attempt to Quit	
Yes	159 (39.6%)
No	242 (60.4%)
Marital Status	
Married	294 (73.5%)
Divorced/Separated	29 (7.2%)
Widowed	22 (5.5%)
Single, never married	45 (11.3%)
Other	10 (2.5%)
Highest Educational Level	
Incomplete secondary educa	16 (4.0%)
Complete secondary educat	97 (24.3%)
Vocational	132 (33.0%)
Higher Education	155 (38.8%)
Self-Rated Health	
Excellent	40 (10.2%)
Very Good	61 (15.5%)
Good	131 (33.3%)
Fair	147 (37.4%)
Poor	14 (3.6%)
Monthly Household Income	
Below 100,000 dram	96 (24.4%)
100,000-300,000 dram	193 (49.1%)
300,000-500,000 dram	74 (18.8%)
More than 500,000 dram	30 (7.6%)

## Results

### Demographics

A total of 811 people met eligibility criteria and 405 (49.9%) consented to participate in the full survey. 99.0% (n=401) of these surveys were considered complete (> 95%) and included in the analysis. Full demographic data is available in **Table 1**. Ages skewed slightly younger but approximately one-third of men were each in their fifth (38.7%), sixth (33.0%) and seventh (28.3%) decades of life.

On average, respondents smoked 22 cigarettes per day, or slightly over one pack per day. There was a significant range in responses from 2 to 100 cigarettes per day. Respondents had smoked an average of 25 years and almost 40% had at least one prior quit attempt. Despite over a third (38.7%) being younger than the USPSTF-recommended age for lung cancer screening (50-80 years), already two-thirds (66.3%) of those surveyed reported smoking 20 pack-years or more. Most men were married (73.5%), had completed secondary, vocational or higher education (96.0%), and had a monthly income of 100,000-300,000 dram (49.1%), roughly comparable to the average Armenian monthly nominal wage of approximately 200,000 dram ($491 USD) (18). Of note, there was a statistically significant negative correlation between age and measures of both income and self-rated health; older respondents, on average, had lower incomes and poorer self-rated health.

### Perceived risk

**Figure 3:**
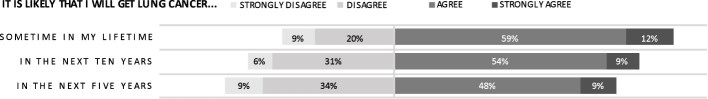
Perceived risk.

Nearly three-quarters of respondents (71.0%) agreed they were at risk for lung cancer over their lifetime (**Figure 3**). However, a lower proportion felt the proximity of this risk: 20% fewer agreed that they faced this risk in the next 5 years. A lung cancer diagnosis was felt to carry a high risk of death by 81.3% of respondents. Notably, more than 50% of respondents did not connect tobacco use with increased lung cancer risk: 40.5% of respondents felt that they had “about the same risk” of lung cancer as non-smokers and an additional 10.8% felt that their risk of lung cancer was actually lower than non-smokers. When data on risk perception was further stratified by age group, there were no clear trends.

### Perceived benefits

**Figure 4:**
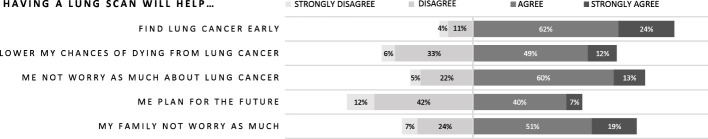
Perceived benefits.

Most understood that a lung scan could help detect cancer earlier (85.7% of respondents agreed or strongly agreed), but almost 30% fewer felt that this would decrease their chances of dying from lung cancer (**Figure 4**). Less than half of respondents felt a lung scan would help them plan for the future, indicating a sense of diagnostic futility. However, a lung scan - presumably negative - might help their family worry less (69.7%).

### Perceived barriers

**Figure 5:**
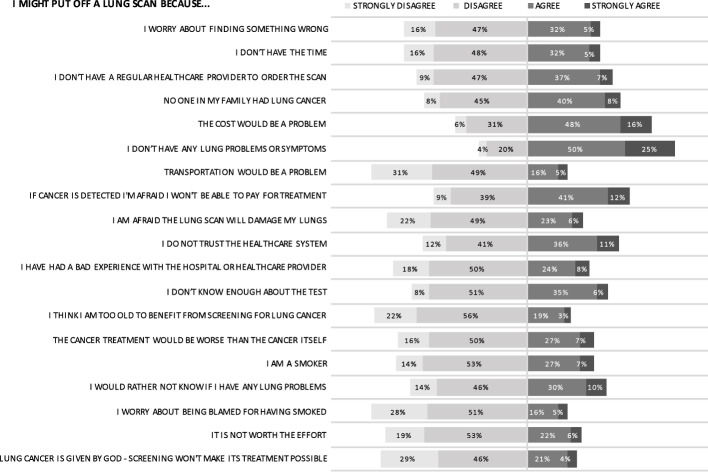
Perceived barriers.

The most commonly agreed-upon barriers to undergoing a lung scan were absence of symptoms, cost of scan, and paying for treatment (**Figure 5**). Higher education, which was correlated with higher income, led to decreased perception of financial barriers for both screening and treatment. Despite almost three-quarters of respondents agreeing they were at risk for lung cancer over their lifetime, the understanding of this risk was nuanced: 75.5% of respondents might put off a lung scan in the absence of symptoms and 47.7% of respondents might put off a lung scan because they did not have any family members with lung cancer. Furthermore, almost one in two respondents (46.9%) indicated a lack of trust in the healthcare system. Respondents generally felt strongly that transportation would not be an impediment to screening.

### Self-Efficacy

**Figure 6:**
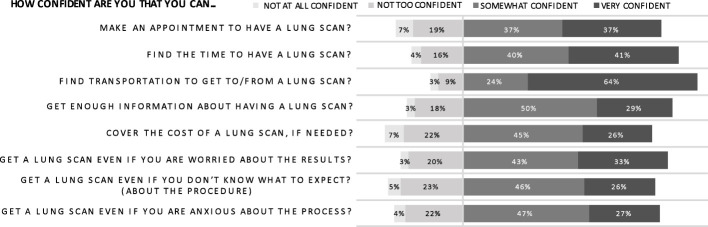
Perceived self-efficacy.

Most respondents felt at least “somewhat confident” that they could handle the logistics of a lung scan if needed (**Figure 6**). In particular, they were “confident” or “very confident” that they could find the time (80.8%) and transportation (88.0%) to undergo a scan. However, financial concerns were again highlighted in this category; there was less confidence about covering the cost of a scan with only 26.0% of respondents feeling “very confident.” Respondents were also more apprehensive about obtaining adequate scan information. Age ultimately did not predict mean self-efficacy scores but, for many individual self-efficacy questions, older age correlated with decreased confidence in ability to coordinate scan logistics. Specifically, decreased confidence secondary to anxiety and concern regarding scan results correlated with increasing age. These findings may indicate a more deep-seated trepidation amongst older men regarding lung cancer than evident in the construct of perceived risk, where increasing age did not correlate with perceived risk.

Analysis showed that there were several statistically significant correlations between the target constructs. Specifically, there was a statistically significant positive correlation between perceived benefits and self-efficacy, r =.25, p <.05 and perceived risk and self-efficacy, r =.15, p <.05. There was a statistically significant negative correlation between perceived benefits and perceived barriers, r = -.15, p <.05 and perceived barriers and self-efficacy, r = -.56, p <.05.

The results from stepwise multiple regression analysis showed that several demographic variables significantly predicted screening attitudes. Specifically, respondents’ perception of the benefits of lung cancer screening was significantly predicted by self-rated health, R^2^ = .01, *F* (1, 373) = 4.52, *β* = .12, p <.05. Perceived barriers was significantly predicted by monthly household income and education level, R^2^ = .12, *F* (1, 368) = 24.1, *β* (monthly household income) = -.27, *β* (education level) = .15, p <.05. Self-efficacy was significantly predicted by household income and education level, R^2^ =.09, *F* (2, 374) = 18.5, *β* (monthly household income) = -.2, *β* (education level) = .18, p <.05.

## Discussion

This study elucidates key health beliefs amongst Armenian male smokers that could both promote and impede lung cancer screening participation. In light of the significant mortality benefit, international organizations, such as the European Union, recommend nations begin planning to implement lung cancer screening using LDCT (19). However, even where screening is available, there has been low uptake. Organized lung cancer screening is part of the United States’ national cancer prevention strategy with mandated insurance coverage (20), yet only 5-6% of eligible individuals undergo screening (21, 22). To maximize the mortality benefit of lung cancer screening, it is crucial to understand the potential screening barriers, particularly individual beliefs that predict screening uptake (16).

This survey in particular highlights an urgent need for further tobacco education in Armenia. Over half of respondents felt that they had an equivalent or lower lung cancer risk than non-smokers, demonstrating concerning ignorance of the strong causal relationship between smoking and negative health effects. Smokers have 20 times the risk of lung cancer compared to non-smokers (23) and heavy smoking is prevalent in Armenian men (7). Almost two-thirds of survey respondents had already met the 20-pack year USPSTF screening criterion.

Participants’ perceptions and beliefs regarding lung cancer screening indicate that further education about the goal of screening is also needed. Over three-quarters of respondents might put off a lung scan in the absence of symptoms, yet screening serves to identify asymptomatic, early-stage disease. Although most survey respondents agreed that screening facilitates earlier cancer detection, an equivalent proportion did not believe that this could prevent lung cancer death. Screening with LDCT offers an effective mechanism to significantly reduce premature death from lung cancer, largely by stage migration, enabling the majority of cancers to be detected at stage I when the cancer is readily treatable (and typically asymptomatic) (12, 13). Later-stage lung cancer treatment options are limited and usually prohibitively expensive with significant toxicities, particularly in LMICs (24). The cognitive disconnect between early detection and reduction in cancer mortality highlights an opportunity to reverse fatalistic beliefs and to educate Armenians on the curability of early-stage cancer to empower citizens to demand better care.

To some extent, fatalistic attitudes elucidated by the survey probably reflect the current availability and quality of early-stage cancer diagnosis and treatment in Armenia. Inconsistent use of internationally-approved histopathologic protocols often results in confusion and re-biopsy. Only 42% of the WHO’s essential chemotherapeutics are registered as essential medications in Armenia with often prohibitive costs. Radiotherapy is centralized in Yerevan and poor utilization of immobilization devices limits therapeutic value and increases adverse effects (7).

Many of the other beliefs expressed in this survey, including concerns about trust and cost, also likely emanate from realities of the Armenian healthcare system. In a telephone survey on the Armenian healthcare system, 79% of Armenians reported they did not trust the government and 45% indicated they were dissatisfied with the healthcare system (25). Almost half of respondents felt equal access was “bad” or “very bad” and nearly one-third of respondents had not used the healthcare system in the last month due to inability to pay (25). The predictive power of education and income on perceptions of screening barriers and self-efficacy in this study further highlights these concerns. In a country where 84% of healthcare expenditures are out-of-pocket (and often catastrophic) despite health system reforms, such findings are unsurprising (25). To avoid exacerbating existing socioeconomic disparities in healthcare access in Armenia, cost, trust and other socioeconomic factors influencing screening behaviors would need to be carefully addressed in any organized screening program.

### Next steps

Knowledge should be examined further to guide future education and mass media campaigns. Although this survey hinted at significant knowledge gaps among end-users (patients), it would also be important to characterize the knowledge and attitudes of primary care providers in a country where only 30% of primary care providers addressed the harms of smoking in the last year (26). The WHO recommends that Armenia increase anti-tobacco media campaigns and access to tobacco cessation resources (10). Implemented together, tobacco cessation and lung cancer screening have positive synergistic effects and even greater magnitudes of cost-saving (27). One large, multicenter randomized controlled trial demonstrated that 40% of screened patients had quit smoking at seven years, compared to 5% in the general population, highlighting screening as a teachable moment and opportunity for intervention (11). Lung cancer screening could be a valuable platform for promoting tobacco cessation in Armenia.

Cost and payment are particularly important considerations in under-resourced settings. Although studies in China and India have shown that LDCT is an equally effective lung cancer screening modality in middle-income countries, no cost-effectiveness analyses for this income bracket have been published to-date despite the elevated burden of disease (8). Cancer cost Armenia about 2% of its gross domestic product in 2017, not including costs of absenteeism and presenteeism, with the vast majority (90%) of that cost due to premature death, not healthcare expenditure (4, 10, 28). Screening with LDCT is known to be cost-effective for high-income countries (8). Even though LDCT is more expensive than other screening modalities, the number needed to screen to prevent one lung cancer death is relatively low (320) compared to other cancers (e.g. 2000 mammograms to prevent one breast cancer death) (12, 29). Other screening programs in LMICs have been successfully funded through price discrimination (i.e. charging those who can pay) and public-private cost sharing (30). In Armenia, the cost of LDCT screening could be further offset by removing non-evidence-based screening exams from the current national screening strategy and by developing tailored screening criteria and intervals (26, 31).

Finally, a complete evaluation of the required resources for both screening and appropriate treatment of lung cancer in Armenia is needed. Armenia has at least seven computerized tomography scanners per 100,000, four thoracic surgery programs, and relatively up-to-date medical oncology, but it is unclear if there is the capacity to safely perform all steps along the continuum of diagnosis to treatment of lung cancer (7). Given the potential harm of screening without proper staging and treatment, further characterization and strengthening of the continuum is needed prior to piloting any organized screening. Formulating a clear and effective national cancer control plan will be an important part of Armenia’s effort to tackle the growing burden of lung cancer and other NCDs.

## Limitations

This study has several potential limitations. There may have been important unidentified differences between men who initially agreed to undergo the survey and those who declined. The study generalizability is likely limited to urban men. For example, transportation was generally not perceived as a barrier amongst respondents, but the majority of health services are concentrated in Yerevan. Compared to other nations, though, Armenia is relatively small and homogenous with most towns within a three-hour drive. The Nubarashen municipality, a lower-income area of Yerevan representing 1% of Yerevan’s population, was not captured in this survey but it is unlikely that the lack of respondents from this municipality significantly affected the data. Regarding the statistical analysis, the use of forward stepwise linear regression has known limitations, including overfitting, bias, and model instability. Model instability, where small variations in the data can lead to the selection of distinct predictor variables and models, is particularly pronounced when predictor variables are highly correlated, although our data was evaluated for multicollinearity upfront. In short, the ability of the aforementioned independent variables to predict the median scores for the outcomes of perceived risk, benefits, barriers, and self-efficacy may be uncertain, and these results may not be generalizable. Finally, in this study, although education and income were evaluated, knowledge, an important factor in health beliefs and screening behavior, was not thoroughly assessed.

## Conclusion

This study sought to assess the attitudes and beliefs of male smokers towards lung cancer screening in Armenia using an adapted, previously-validated version of the EHBM. Although many of the respondents’ beliefs likely stem from realities of the Armenian healthcare system, this study also uncovered important knowledge gaps and concomitant educational opportunities. While these knowledge gaps warrant further research, this research should not delay robust national education on tobacco use, the nature of asymptomatic disease, and the role of screening.

## Data availability statement

The raw data supporting the conclusions of this article will be made available by the authors, without undue reservation.

## Ethics statement

The studies involving human participants were reviewed and approved by 1) Office of Human Research Protection Program at the University of California, Los Angeles (IRB#21-000784) 2) Ethics Committee at Yerevan State Medical University. Written informed consent for participation was not required for this study in accordance with the national legislation and the institutional requirements.

## Author contributions

NC: Conceptualization, literature search, interpretation of data, project administration, writing of the original draft. HT: Literature search, interpretation of data, writing of the original draft, visualization (figures), critical revision and editing of the paper. TG: Data curation, validation of all data provided in the paper, data analysis, critical revision and editing of the paper, validation of translation. AL: Study design, validation of translation, supervision and mentorship, critical revision and editing of the paper. JD: Study design, supervision and mentorship. MH: Translation, data collection, critical revision and editing of the paper. SS: Conceptualization, interpretation of data, project administration, supervision and mentorship, critical revision and editing of the paper. All authors contributed to the article and approved the submitted version.
